# Dietary sugars: their detection by the gut–brain axis and their peripheral and central effects in health and diseases

**DOI:** 10.1007/s00394-014-0776-y

**Published:** 2014-10-09

**Authors:** Melissa Ochoa, Jean-Paul Lallès, Charles-Henri Malbert, David Val-Laillet

**Affiliations:** 1 INRA, UR1341 ADNC, Domaine de la Prise, 35590 Saint-Gilles, France; 2 INRA, US1395 Ani-Scans, Domaine de la Prise, 35590 Saint-Gilles, France

**Keywords:** Dietary sugars, Gut–brain axis, Sugar sensing, Eating behaviour, Reward circuitry

## Abstract

**Background:**

Substantial increases in dietary sugar intake together with the increasing prevalence of obesity worldwide, as well as the parallels found between sugar overconsumption and drug abuse, have motivated research on the adverse effects of sugars on health and eating behaviour. Given that the gut–brain axis depends on multiple interactions between peripheral and central signals, and because these signals are interdependent, it is crucial to have a holistic view about dietary sugar effects on health.

**Methods:**

Recent data on the effects of dietary sugars (i.e. sucrose, glucose, and fructose) at both peripheral and central levels and their interactions will be critically discussed in order to improve our understanding of the effects of sugars on health and diseases. This will contribute to the development of more efficient strategies for the prevention and treatment for obesity and associated co-morbidities.

**Results:**

This review highlights opposing effects of glucose and fructose on metabolism and eating behaviour. Peripheral glucose and fructose sensing may influence eating behaviour by sweet-tasting mechanisms in the mouth and gut, and by glucose-sensing mechanisms in the gut. Glucose may impact brain reward regions and eating behaviour directly by crossing the blood–brain barrier, and indirectly by peripheral neural input and by oral and intestinal sweet taste/sugar-sensing mechanisms, whereas those promoted by fructose orally ingested seem to rely only on these indirect mechanisms.

**Conclusions:**

Given the discrepancies between studies regarding the metabolic effects of sugars, more studies using physiological experimental conditions and in animal models closer to humans are needed. Additional studies directly comparing the effects of sucrose, glucose, and fructose should be performed to elucidate possible differences between these sugars on the reward circuitry.

## Introduction

Dietary sugar intake, in the form of sucrose or high-fructose corn syrup (HFCS), has dramatically increased and correlates with a rise in obesity, metabolic syndrome, and diabetes [1]. Because a broad range of physiological, behavioural, and neurological variables influences food choices and eating behaviour, it is difficult to understand the mechanisms of eating behaviour and their alterations. The hedonic value of highly palatable foods and their wide availability can override the physiological mechanisms related to energy homeostasis [2, 3]. The hedonic reward value of food is closely linked to the sensory perception of food (including food taste, odour, and texture) and refers to the driving force behind the motivation to eat. The nutrient detection by the gut is mainly controlled by enteroendocrine (EE) cells and might activate a cascade of physiological phenomena, including endocrine regulations (e.g. insulin, leptin, glucagon-like peptide-1 or GLP-1, secretion), inhibition of gastric emptying, inhibition of food intake [4], stimulation of intake [5] as well as psychobehavioural responses [6].

Dietary sugar overconsumption might provoke deleterious effects at both central and peripheral levels, including alterations in (i) the regulation of secretion of satiety peptides and neuropeptides [7, 8]; (ii) gut permeability leading to low-grade inflammation and liver disease [9]; (iii) blood–brain barrier (BBB) permeability [9]; (iv) the endocannabinoid [10], opioid [8], and mesolimbic dopaminergic systems, as well as (v) brain structures involved in reward processing [11]. Both drugs and food have powerful reinforcing effects partly mediated by dopamine increases in the limbic system that, under certain circumstances or in vulnerable individuals, could overwhelm the brain’s homeostatic control mechanisms [11], but the plausibility of sugar addiction and its role in obesity and eating disorders in humans is still a subject of controversy [12].

Much of the research on the effects of dietary sugars on health has recently focused on fructose, given the striking parallel increases in obesity and in fructose intake over the past decades [13]. These studies have found important fructose-induced health disturbances that are different from those provoked by glucose or sucrose. Most of fructose intake in diets originates from sucrose (containing 50 % fructose and 50 % glucose) and soft drinks containing high-fructose corn syrup (HFCS) (range 47–65 % fructose, and 53–35 % glucose) [14]. An estimate of the consumption of HFCS from beverages indicates a daily range between 132 and 316 kcal for Americans aged over 2 years [13], and patients with non-alcoholic fatty liver disease (NAFLD) consume twofold more calories from HFCS from beverages than healthy patients (365 vs. 170 kcal/day) [15]. In the United States, average fructose consumption from sugar-sweetened beverages has increased from 37 to 49 g/day during the last 30 years (+0.4 % per year) [16]. The increase in fructose consumption is synonymous with increased energy intake. Thus, it is not clear whether the fructose-induced metabolic disturbances observed in human and animal studies are due to fructose itself or the associated increase in energy intake. Moreover, since fructose and glucose intake may vary simultaneously, this raises the consideration that other dietary sugars (e.g. sucrose and glucose) might also contribute to the development of obesity and associated co-morbidities.

In fact, there are controversial findings on metabolic effects between the different sugars (i.e. glucose, fructose, and sucrose). While some studies have disclosed significant differences between these sugars, other studies have found small or no difference. For example, in overweight or obese humans, intake of a fructose-sweetened beverage led to a significant increase in visceral adipose tissue, hepatic de novo lipogenesis, and postprandial triglycerides compared to subjects offered a glucose-sweetened beverage [17]. In lean and obese subjects, de novo lipogenesis increased to the same extent after overfeeding with glucose and sucrose [18]. Both high-glucose and high-fructose diets stimulated lipogenic gene expression in rodents [19].

Most of the studies on the effects of sugars on health and disease, at both peripheral and central levels, have been performed in rodents, and studies are missing in humans or other animal models closer to humans, such as the pig model. Given that human studies are limited due to ethical considerations, future studies should privilege the use of animal models that closely resembles humans.

On the other hand, there is an impressive number of studies available concerning the effects of dietary sugars, using different experimental paradigms, with different approaches, animal models, oral intake doses, in the form of sugar solutions or added in the diet, or peripheral or central administrations. Therefore, it becomes extremely difficult to interpret and find a definite conclusion on these effects. In this context, we considered essential to gather all the information available to give a global view of the current research in this domain, in order to highlight the need to reformulate the questions and approach to these questions, under similar conditions between studies, and using integrated approaches, from the molecular to the behavioural level.

The main goal of this review was to provide an overview of the impact of different dietary sugars on peripheral and central functions. It will gather (i) results from studies regarding the effects of sucrose, glucose, and/or fructose on metabolism, eating behaviour, and brain responses; (ii) current available data comparing the effects of these sugars, at both peripheral and central levels. It will also propose some clues and hypotheses for future research perspectives regarding the effects of these sugars, with special focus on fructose and glucose.

## The pig model in biomedical research

Even though this review synthetises data from different animal models and humans, we wanted to dedicate a short section to the presentation of the pig model, which is of particular interest in nutrition and neurosciences. Pigs have emerged as an ideal model for nutritional and biomedical research because of their anatomical and physiological similarities to humans [20–22], as well as blood chemical and biochemical characteristics, plasma hormone levels, and energy metabolism [23]. Pigs are able to distinguish the palatability of different diets, and they have a high innate preference for sweet taste [24] and a strong appetite for sugar solutions [25]. The digestive system of pigs has anatomical differences with that of humans; however, the physiology of digestion is essentially similar. Swine are true omnivores. In spite of the anatomical differences, the pig has been used extensively as a model of digestion in connection with nutrition (and determination of food value) of the pig and for studying human digestive phenomena. The metabolic functions, intestinal transit times, and characteristics of nutrients absorption have made them useful in basic nutritional research. Other specific functional characteristics of swine that relate directly to humans include ion transport and motility, neonatal development of the gastrointestinal tract, and splanchnic blood flow characteristics. Like humans, these physiological characteristics of the gastrointestinal tract are probably due to the omnivorous diet they consume, unlike that of carnivores, ruminants, rabbits, and rodents [26]. Similar to carbohydrate (sucrose and starch) digestion in humans [27], it was shown in non-anaesthetised mature pigs following the intake of different sugar-containing meals (with glucose, sucrose, lactose, or maize starch) that the absorption pattern was different for each sugar. The kinetics of appearance of glucose and of sucrose hydrolysis products in the portal blood were faster for glucose and sucrose than for sugars resulting from maize starch hydrolysis [23].

Recent studies have shown the convenience for the use of pig model in brain imaging, behavioural, and physiological effects of obesity induced by highly palatable diets [20, 22, 28, 29]. Compared to the rodent brain, the pig brain more closely resembles the human brain in terms of anatomy and biochemistry, which associates the pig with a higher translational value. Several brain disorders have been fully or partially modelled in the pig, and this has further spurred an interest in having access to behavioural tasks for pigs and in particular to cognitive tasks. Cognitive testing of pigs has been conducted for several years in animal science, but it has only recently received interest in the wider neuroscience community. Several behavioural tasks have successfully been adapted to the pig, and valuable results have been produced [30].

Aside of having similar brain structures to humans, the pig might develop metabolic disorders observed in humans (excessive fat deposition, diabetes, atherosclerosis, hypertension) [31]. Taken together, these data position the pig model as a valuable model for biomedical studies in nutrition and neuroscience. Therefore, future studies on the effects of dietary sugars on health and disease should favour the use of the pig model in order to extrapolate data to humans and propose modifications in the nutritional recommendations for humans.

## Effects of dietary sugars on gut microbiota, intestinal barrier, and liver

Gut microbiota operates like a metabolic organ, influencing nutrient availability and uptake, energy homeostasis, and the control of body weight. Diet composition may strongly influence changes in the microbiota, which in turn, when subjected to deleterious nutritional environment, might affect intestinal permeability and result in low-grade inflammation, obesity, and associated chronic metabolic diseases such as NAFLD, dyslipidaemia, and insulin resistance [9, 32].

Increases in gut permeability, low-grade endotoxemia (provoked by increased plasma lipopolysaccharides—LPS), and hepatic lipid accumulation have been reported in animal models of obesity induced by high-fat or high-fructose diets. A high-fructose diet has been associated with hepatic and extra-hepatic insulin resistance and obesity-related metabolic disturbances through a mechanism implicating gut microbiota and its effects on intestinal permeability [9].

### Liver disease and inflammation

The hepatic manifestation of the metabolic syndrome is NAFLD, starting from simple steatosis and ending as liver cirrhosis. Dietary sugar intake might participate to NAFLD pathogenic history, and the type of sugar (e.g. fructose) may affect the development of the disease [33]. Consistent evidence has demonstrated the implication of fructose or HFCS in the development of NAFLD in several animal models [17, 33–38]. The effects of dietary sugars, especially fructose, on the development of hepatic steatosis, liver damage, and other features of the metabolic syndrome found in several animal models are presented in Table 1.

**Table 1:** Effects of dietary sugars intake on hepatic steatosis, liver damage, and various features of the metabolic syndrome in several animal models and in humans

Dietary intervention	Duration	Hepatic steatosis/liver injury	Plasma or hepatic measurements	Other effects	Model	Animal studies
20 % HFCS in maternal diet 20 % HFCS + MSG	9 Months	Hepatic steatosis	↑ Hepatic and serum TG, serum FFA	↑ SREBF2 expression	C57BL/6 J mice	Collison [39]
High-fat/high-fructose diet: (A) 8 % trans-fat, 20 % HFCS (B) trans-fat (8 %), HFCS ^a^ (20 %), 1 % MSG	9 Months	↑ ALP, ALT	↑ Serum creatinine, cortisol	↓ Serum leptin and fasting insulin	Domestic cat (male, n = 18)	Collison [40]
(A) Trans-fat (9 %) +HFCS (24 %) (B) Trans-fat (9 %), HFCS^a^ (24 %), 1 % MSG	9 Months	Fibrous expansion ↑ Markers of hepatic fibrosis, angiogenesis, hepatocellular carcinoma	↑ ALP and ALT Creatinine and cortisol (A)		Domestic cat (male, *n* = 18)	Collison [41]
30 % Glucose solution 30 % Fructose solution	8 Weeks	Lipid accumulation	↑ Endotoxin levels in portal blood TNFα higher with fructose than glucose	Antibiotics prevented some effects of fructose	C57BL/J6 mice	Bergheim [42]
*Fructose + antibiotics*						
30 % Fructose solution (F) Tap water (C) 30 % Fructose solution + antibiotics polymyxin B and neomycin (AB)	8 Weeks	Hepatic fat accumulation and TNFα and iNOS mRNA expression in liver with F suppressed by AB	↑ Endotoxin TLR4 receptors, suppressed by AB	↓ Tight junction occludin in duodenum (F)	C57BL/J6 mice	Wagnerberger [43]
30 % Fructose solution	8 Weeks	Induction of hepatic steatosis and inflammation	↑ ALT levels	↑ Hepatic lipidperoxidation, phospho-ΙκΒ, NF- κB and TNFα	C57BL/J6 mice	Spruss [44]
Fructose group: 10.5 % fat 20 % fructose Atherogenic diet: 20 % fructose 46 % fat, 2 % cholesterol, 0.7 % choline	24 Weeks	Fructose diet: normal liver histology Atherogenic diet: abnormal liver histology with microvesicular steatosis and fatty Kupffer cells but not fibrosis		Fructose group: ↑ body weight, hypertension, and insulin resistance Atherogenic diet: metabolic syndrome	Osabaw minipig	Lee [31]
Standard chow diet: (S) 41 % Starch, 5 % sugar, 4.5 % fat, iron (120 mg/kg) Fructose-enriched diet (F) 60 % fructose, 5 % fat + Iron (5 mg/kg)	5 Weeks	F: Higher liver lipids, TG, and cholesterol than S group Mild-to-moderate deposition of macrovesicular and microvesicular fat Similar hepatic iron concentration in both groups	F: ↑ TG, insulin, HOMA score	F: ↑ blood pressure	Sprague–Dawley rats *n* = 49	Ackerman [183]

Dietary intervention	Duration	Hepatic steatosis/liver injury	Plasma or hepatic measurements	Other effects	Recruitment criteria	Human studies
Usual ad libitum diet + fructose (*n* = 16) or glucose (*n* = 15) sweetened beverages at 25 % of energy requirements	10 Weeks	Not investigated	Fructose group: Fasting plasma MCP-1, PAI-1, and E-selectin Postprandial PAI-1		BMI: 25–35 kg/m^3^ Healthy,40–72 year	Cox [184]
Usual ad libitum diet + fructose (n = 17) or glucose (n = 15) sweetened beverages at 25 % of energy requirements	10 weeks	↑ Fractional hepatic de novo lipogenesis only in fructose group	Fructose group: ↑ Plasma lipid and lipoprotein ↑ Postprandial TG, lipoproteins, and LDL cholesterol Glucose group: ↑ Fasting TG and FFA in glucose group ↑ Postprandial lipoprotein lipase activity	↑ Body weight, fat mass in both groups ↑ Abdominal and visceral adipose tissue only in fructose group ↓ insulin sensitivity only in fructose group	BMI: 25–35 kg/m^3^ Healthy, 40–72 year	Stanhope [17]

*HFCS* high-fructose corn syrup, *TG* triglycerides, *SREBF2* sterol regulatory element-binding transcription factor 2, *ALP* alkaline phosphatase, *ALT* alanine aminotransferase, *TNFα* tumour necrosis factor alpha, *iNOS* inducible nitric oxide synthase, *AB* antibiotics, *NF- κΒ* nuclear factor κΒ, *TLR* Toll-like receptors, *HOMA* homeostatic model assessment, *MCP-1* monocyte chemoattractant protein 1, *PAI-1* plasminogen activator inhibitor type 1, *FFA* free fatty acids

It seems that HFCS or fructose exposure is able to induce hepatic steatosis, liver dysfunction, hepatic fibrosis as well as several features of the metabolic syndrome and inflammation in rodents and cats (e.g. [39–44]). However, in other species such as pigs or in humans, this concept remains incompletely clear. Data obtained from humans [45] and Osabaw minipigs [31] suggested that it is the association between high-fructose intake with other components in the diet, such as glucose, sucrose, fat, and cholesterol, responsible for the development of the metabolic syndrome and liver steatosis, rather than high-fructose intake itself. The approach used in several human studies where fructose daily intake pattern is assessed in patients with previously established hepatic steatosis [33, 46, 47] is not the best way to evaluate fructose as a risk factor for NAFLD. This question should be addressed in a more controlled experimental paradigm where dietary intake is closely monitored. To our knowledge, one of the few human studies that has assessed the effect of glucose- or fructose-sweetened beverages on the development of hepatic de novo lipogenesis under controlled conditions is the one performed by Stanhope et al. [17]. However, this study did not confirm the presence of hepatic steatosis using standard diagnostic methods such as MRI, CT scan, or liver biopsy. Thus, studies in humans are needed to investigate the effects of dietary sugars on the development of hepatic steatosis under controlled experimental conditions.

Taken together, studies with rodents and cats suggest that fructose induces liver damage, in part through mechanisms involving intestinal bacterial overgrowth, increased intestinal permeability, inflammation, and metabolic endotoxemia. However, underlying mechanisms explaining how fructose leads to bacterial overgrowth, inflammation, and increases in intestinal permeability remain poorly understood. Additional studies are necessary to further explore this hypothesis in humans. Since it is difficult to achieve controlled experimental conditions in humans, for ethical reasons, studies in animal models closer to humans, e.g. pigs [20, 28, 31], are a valuable approach that allows close monitoring of dietary interventions. If similar mechanisms occur in humans and pigs, novel strategies including low-fructose diets might be considered for the prevention/management of NAFLD. However, there seems to be substantial differences between rodents or cats and humans or pig studies. Thus, in the absence of clear evidence for a detrimental role for fructose, there is no justification for replacing it with other dietary sugars such as glucose or sucrose in human diets for the prevention of hepatic steatosis.

## Effects of dietary sugars on the regulation of food intake

The regulation of food intake and energy homeostasis is achieved by a complex network communication between the periphery (e.g. gut, liver, stomach, pancreas, and adipose tissue) and the brain. This regulation has been extensively reviewed already (e.g. [48–51]). The different molecular structures of dietary sugars might result in different gastrointestinal peptide secretion profiles, leading to different metabolic and endocrine effects at both peripheral (e.g. gut and liver) and central (e.g. hypothalamus) levels [52–54]. It has been shown that fructose, compared to glucose intake, produces smaller increases in plasma glucose and circulating satiety hormones, i.e. insulin, leptin, glucagon-like peptide-1 (GLP-1), peptide tyrosine tyrosine (PYY), and attenuates postprandial suppression of ghrelin [37, 55–59]. This suggested an endocrine mechanism by which fructose might induce a positive energy balance and weight gain. A possible explanation of smaller increases in satiety hormones by fructose could be the lower expression of GLUT5 in β-cells [60] or lower absorption rates in the intestine [61]. In addition, it was found that central administration of fructose provokes feeding in rodents, whereas centrally administered glucose promotes satiety [54, 62]. These data together with parallel increases between fructose intake and obesity development [13] have led to the ‘fructose hypothesis’ which postulates that fructose, compared to glucose, may stimulate food-seeking behaviour, food intake, and body weight gain. However, the proposed effect of fructose on the induction of feeding is the subject of debate since this concept has not been replicated in rodents, and there is little evidence linking these phenomena in humans [56, 58, 59, 63, 64]. The few studies linking fructose consumption with increased body weight compared fructose *versus* an artificial sweetener [65], evaluated 60-g fructose supplementation but did not compare it *versus* another sugar [66], or used higher doses of fructose compared sucrose [67]. Even others found no substantial differences in endocrine and metabolic effects after consumption of sugar-sweetened beverages with HFCS, sucrose, fructose, and glucose in humans [68] or in body weight and food intake [69] or found greater increase in body adiposity with sucrose than with fructose solutions [70]. Moreover, it appears that fructose orally ingested may cross the BBB to a small extent compared to glucose, which may have two opposite implications: 1) the limited fructose access to glucose-sensing neurons could contribute to the deregulation of food intake and energy balance, or 2) fructose might have no effect at all on appetite regulation due to the lack of fructose transport to the brain. These hypotheses need to be investigated and clarified in future studies. The purpose of this section was to present available data on the main enteric and cephalic detection processes of dietary sugars, in association with satiety peptides, neuropeptides secretion, and neuronal activity, and to discuss their effects on food intake.

### Oral and enteric detection of dietary sugars: sweet taste receptors and sugar transporters

Peripheral sweet taste and sugar detectors are key regulators of feeding behaviour and energy homeostasis. Taste-signalling mechanisms identified in the oral epithelium also operate in the gut and play a role in both sugar detection and regulation of intestinal and pancreatic hormone secretion [71]. There are two main groups of sugar detectors: members of the G-protein-coupled receptors (GPCR) family, and sugar transporters (e.g. GLUT2, GLUT5, sodium-dependent glucose co-transporter 1 (SGLT1), and GLUT8). Enteroendocrine cells directly sense sugars via GPCR, including the sweet taste receptors of type 1, T1R. These receptors have also been implicated in sweet taste preferences [72].

The T1R2/T1R3 heterodimers form sweet taste receptors that recognise several natural and synthetic sweeteners. The initial step in taste recognition occurs on the apical surface of taste receptor cells, within taste buds of the tongue and palate [73]. The regulation of taste sensitivity by appetite peptides at the level of taste bud cells in the tongue as well as in enteroendocrine cells of the taste epithelium may be important in the control of eating behaviour and the regulation of energy homeostasis [74]. However, this concept remains unclear since it was found that knockout (KO) mice (P2X2/P2X3) with adenosine triphosphate taste cell signalling deficits show relatively normal food intake and body weight. They also develop strong preferences for non-taste nutrients by the post-oral actions of these nutrients [75]. Furthermore, much of the research on appetite peptides and taste detection in the mouth has been performed at the cell/neuron level; however, little empirical evidence exists to date for these peptides impacting taste function at the level of the mouth. Therefore, more studies should be performed in large animal models to clarify these concepts.

Appetite regulatory peptides, such as leptin, endocannabinoids, GLP-1, glucagon, oxytocin, insulin, cholecystokinin (CCK), neuropeptide Y (NPY), and vasoactive intestinal peptide (VIP), modulate taste sensitivity at the level of oral sweet taste cells [74]. Leptin selectively suppressed sweet taste responses of cells isolated from circumvallate papillae from non-diabetic mice, but not in diabetic *db/db* mice. This indicated that the effect of leptin on sweet taste sensitivity is mediated by the leptin receptor expressed in these cells [76]. The T1R3 subunit is co-expressed with the leptin receptor in both fungiform and circumvallate taste bud cells, and leptin suppresses sweet taste sensitivity in mice by affecting responsiveness of T1R3-expressing taste cells via the leptin receptor. Therefore, leptin may play an important role in the regulation of sweet taste sensitivity in the tongue, besides its central actions on food intake [77].

Co-expression of GLP-1 with taste-signalling elements such as T1R2, T1R3, and α-gustducin, a Gα_i_ family member associated with taste perception, was found in human intestinal endocrine L cells [78]. These taste-signalling elements mediate the glucose-dependent secretion of GLP-1 and maintain or enhance sweet taste sensitivity via paracrine action [78]. In addition to its intestinal expression, GLP-1 was also found to be expressed in taste cells in mouse circumvallate papillae taste buds; it was co-expressed with α-gustducin and T1R3-expressing sweet taste cells in mouse taste buds, and it was produced in taste buds from lingual extracts in its active form [79]. In contrast to its presence in blood and ileum [80], dipeptidyl peptidase 4 was not found to be expressed in taste buds, suggesting that the half-life of GLP-1 in taste tissue should be high, ensuring sufficient concentrations within the taste bud to stimulate the GLP-1 receptor [79].

HEK-293 cells expressing Gα15 (a phospholipase C-linked G-protein), cotransfected with human and rat T1R2/T1R3, respond to all sweet taste stimuli: sucrose, fructose, galactose, glucose, lactose, and maltose [81]. In the rat, however, the relative lack of T1R2 expression in taste bud cells of the fungiform papillae is consistent with the relative low response to sucrose recorded at the level of the chorda tympani nerve [82] The activation of T1R1/T1R3 and T1R2/T1R3 in rat small intestine by glutamate, glucose, and artificial sweeteners increases the apical expression of GLUT2 and sugar absorption [83, 84]. Given this effect of sweet taste per se to activate T1R2/T1R3 and sugar absorption, it would have been expected that artificial sweeteners might also slow gastric emptying. However, intragastric or intestinal administration of equisweet solutions with artificial sweeteners and/or fructose did not modify glucose absorption rates, plasma glucose, incretin levels, or gastric emptying in humans [85–87] or rodents [88] as a glucose solution did. Collectively, these findings did not support the concept that the sweet taste per se is the principal detection mechanism, responsible for the regulation of gastric emptying, glucose absorption, or incretin release. Therefore, these data are in contrast to previously reported parallels between nutrient-sensing pathways in the oral cavity and gut [89]. It seems that artificial sweeteners may influence the expression of sugar transporters such as GLUT2 [83, 84] and SGLT1 [90], but they may not influence other physiological functions such as gastric emptying or glucose absorption, and their effect on incretin release seems contradictory since some studies reported an effect of artificial sweeteners (sucralose) on GLP-1 secretion from the ileum via T1R3 activation [71], while other studies did not find any effect of artificial sweeteners on incretin levels in humans [85–87] or rodents [88] when compared to glucose effects. Therefore, these data make unclear whether sweet taste receptors are necessary in such gastrointestinal functions.

T1R1/T1R3 and T1R2/T1R3 stimulation leads to the activation of α-gustducin [91]. T1R3 and α-gustducin are necessary for increased stimulation of (SGLT1) by dietary sugars [92]. T1R3 inhibition with lactisole decreased fructose stimulation of human SGLT1, GLUT5, and L-pyruvate kinase mRNA expression, demonstrating the implication of T1R3 in fructose signalling, whereas T1R3 did not control GLUT2 expression and activity [93].

In enterocytes, cell polarity may influence the regulation of sweet taste receptor signalling. TIR2 and T1R3 are located at the basolateral membrane of differentiated enterocytes. Whereas the apical supply of fructose increased GLUT5 mRNA expression, the basolateral supply of sugars increased GLUT2 expression, suggesting that sugars can directly signal from the basolateral membrane [93].

When intestinal luminal glucose concentration is lower than in plasma, glucose is transported by SGLT1 through the apical membrane against the concentration gradient. Dietary fructose is transported across the apical membrane by the facilitative transporter GLUT5. In the basolateral membrane, both glucose and fructose are transported by GLUT2 [94, 95]. At high glucose or fructose concentrations, when SGLT1 and GLUT5, respectively, are saturated, GLUT2 translocates to the apical membrane where it complements SGLT1 and GLUT5 transport capacities. Apical GLUT2 participates in the energy-sensing mechanism [96]. Depending on its relative abundance in the apical and basolateral membranes, it may stimulate sugar signals from intestinal lumen or bloodstream. Chronic exposure to a high-sugar diet promotes increased apical GLUT2 levels, increases glucose absorption, and excessive postprandial excursions [94]. Insulin induces the internalisation of apical GLUT2, a process that is impaired in insulin resistance, contributing to further glucose absorption [97]. Since GLUT2 depends on glucose transport by SGLT1, i.e. it promotes GLUT2 upregulation [98], long-term regulation by SGLT1 may also be reflected in changes in apical GLUT2 [94]. In piglets fed isocaloric diets with variable concentrations of digestible carbohydrates (i.e. sucrose and maize starch), SGLT1 expression remains constant after exposure to diets containing up to 40 % digestible carbohydrate. However, after exposure of >50 % carbohydrate diets, SGLT1 expression is markedly increased. In contrast, under both low- and high-carbohydrate diets, GLUT2 is expressed on the basolateral membrane of pig enterocytes. These results suggest that SGLT1 is the major route for the absorption of dietary sugars across the luminal membrane of swine enterocytes [99]. Moreover, duodenal and jejunal infusions of glucose, fructose, and saccharin induced up-regulation of SGLT1 in mice apparently involving vagal afferents [90]. Altogether, these data suggest that SGLT1 and apical GLUT2 are potential targets for antidiabetic therapy [90, 93, 94]. Duodenal SGLT1 and GLUT5 mRNA expressions and protein levels are substantially increased in diabetic patients. Reduction in plasma glucose in these patients promoted a reduction in both SGLT1 and GLUT5 levels, suggesting that under hyperglycaemic conditions, the absorption of sugars is enhanced [100]. Moreover, postprandial plasma fructose levels are increased in diabetic patients and are associated with the prevalence of diabetic retinopathy [101]. In contrast, in Zucker diabetic rats, mRNA and protein levels of SGLT1, GLUT5, and GLUT2 were unchanged compared to lean controls [102]. This suggests that the Zucker diabetic rat might not be a good model for the study of diabetes since it does not reproduce results observed in humans.

Consumption of a large amount of pure fructose can exceed the capacity of intestinal fructose absorption, resulting in diarrhoea. However, the consumption of glucose along with fructose, as it is usually consumed in beverages and with meals (e.g. when provided as sucrose), appears to enhance fructose absorption. In addition, fructose absorption increases during sustained fructose consumption, suggesting an adaptation to increased fructose intake [37]. GLUT8, expressed only in the intracellular compartment, potentially mediates sugar transport into or out of intracellular organelles [103]. GLUT8 has high affinity for glucose, whereas fructose and galactose compete with glucose transport activity [97]. Its deficiency enhances fructose uptake in cultured Caco2 human intestinal epithelial cells and in jejunum isolated from mice lacking the gene encoding GLUT8. Moreover, mice lacking GLUT8 rapidly develop higher serum fructose concentrations after oral fructose gavage. These effects are possibly mediated by the stabilisation of the dual-specificity glucose/fructose transporter GLUT12 [104]. These data might lead to the speculation that this transporter could, in part, be implicated in fructose malabsorption previously reported when ingested at high levels in humans [105]. Further studies are needed to investigate this hypothesis.

### Enteric dietary sugar sensing and the regulation of peptides secretion

The glucose-dependent secretion of GLP-1 plays a critical role in the regulation of glucose homeostasis. It was shown that T1R3, but not T1R2, affects both incretin secretion from the intestine and insulin secretion from the pancreas [71]. Exposure to glucose, fructose, and sucralose induced GLP-1 secretion from the ileum of wild-type (T1R3^+/+^) but not from T1R3 null mice (T1R3^−/−^). T1R2^−/−^ mice showed normal glycaemic control and partial small intestine glucose-stimulated GLP-1 secretion, suggesting that T1R3 can mediate glucose-stimulated GLP-1 secretion without T1R2 [71]. GLP-2 promotes GLUT2 insertion in the apical membrane, stimulating jejunal fructose transport [106]. SGLT1 and SGLT3 may also be involved in enteric sugar-sensing and hormonal secretion stimulation [107]. SGLT1 triggers glucose-induced secretion of gastrointestinal polypeptide (GIP) from K-cells in the duodenum and jejunum [98]. This in turn stimulates the release of GLP-1 and GLP-2 from L cells located in the ileum [107, 108].

Some evidence has revealed an anorexigenic effect of glucose and an orexigenic effect of fructose through different secretory profile of appetite peptides. For example, glucose and, to a lesser extent, galactose, but not fructose, mannose, or sorbitol, stimulated the release of GIP [52]. Glucose intragastrically infused or orally ingested induced an increase in plasma glucose levels, stimulated insulin, leptin, GLP-1 and peptide tyrosine–tyrosine (PYY) secretion, and reduced ghrelin secretion, while fructose did not substantially affect these hormones [37, 57–59]. However, in one of these studies [58], the amounts of intragastric load of glucose and fructose were different (50 and 25 g/250 mL, respectively), which might have contributed to the observed differences [58].

A possible explanation for the lack of effects of fructose on insulin secretion may be related to lower intestinal mRNA and protein GLUT5 levels compared to GLUT2 levels [109], with the subsequent lower fructose transport compared to glucose. In addition, it was previously shown that glucose absorption rate was higher than that of fructose in Yucatan minipigs [61]. A substantial portion (12 %) of ingested fructose is metabolised to lactate by the gut during absorption, while only 2 % of glucose ingested is metabolised in the gut and almost all of the absorbed glucose appears in the portal vein as glucose. In concordance with lower fructose absorption rates compared to glucose, insulin concentrations were 7.5-fold above basal conditions following glucose intake, compared to threefold following fructose intake [61]. Another possible explanation for the lack of effects of fructose on insulin secretion is the low level of expression of the GLUT5 fructose transporter in β-cells [60]. Taking together these data, i.e. lower expression levels of GLUT5 than GLUT2 in the intestine, lower rate of fructose transport compared to glucose, partial intestinal fructose, but not glucose, metabolism, and the low level of GLUT5 expression in β-cells could explain in part the lower increases in other gut peptides, besides insulin, induced by fructose ingestion, compared to glucose. Further studies are needed to confirm this concept. In this context, since insulin and leptin function as key signals in the central nervous system through the modulation of hypothalamic neuropeptides for the long-term regulation of energy balance, chronic fructose intake could lead to increased calorie intake, thereby contributing to weight gain and obesity [110].

### Cephalic detection of dietary sugars in the regulation of peptide secretion and neuronal activity

Hunger is regulated by the hypothalamus and the dorsal vagal complex in conjunction with an integrated network of limbic brain structures such as the striatum, orbitofrontal cortex, amygdala and insula, which control motivation-reward systems associated with the hedonic drive to eat [97]. The arcuate nucleus of the hypothalamus is an integrator of hormonal and nutrient information to regulate both energy and glucose homeostasis. Brain cells are provided with mechanisms that sense energy availability in the extracellular space, such as an increase in adenosine monophosphate kinase (AMPK) activity in response to an increase in AMP-to-ATP ratio [50].

Glucose-sensing neurons are located in brain areas involved in the control of neuroendocrine function, nutrient metabolism, and energy homeostasis (e.g. hypothalamic arcuate nucleus and the dorsal vagal complex) and also receive direct and indirect neural input from the periphery and from other brain areas that carry information about the characteristics of the ingested nutrients [50]. Glucose-sensing neurons express receptors for and respond to peripheral hormones such as leptin and insulin that convey signals relating to carbohydrate and fat stores. These hormones as well as metabolic substances are transported across the BBB but can also freely diffuse from capillaries to the adjacent median eminence. Anabolic arcuate NPY/agouti-related protein (AgRP) and catabolic proopiomelanocortin (POMC) neurons are metabolic sensors with important roles as regulators of energy homeostasis [111]. Arcuate NPY/AgRP neurons are inhibited by insulin and leptin and, when activated, stimulate food intake (orexigenic), whereas POMC neurons reduce food intake (anorexigenic) and are stimulated by insulin and leptin. Both neuronal subsets project to secondary order neurons located adjacent to hypothalamic areas including the paraventricular nucleus, where anorexigenic neurons are concentrated, and the lateral hypothalamic area, which contains orexigenic neurons. NPY/AgRP neurons also inhibit POMC neurons via synaptic release of the inhibitory transmitter, γ-aminobutyric acid [112]. Through smaller increases in insulin and leptin secretion induced by fructose intake, compared to glucose [55, 59], fructose-containing diets may lead to a lower inhibitory effect of orexigenic neurons NPY/AgRP, as well as a reduced reward value from food [113]. This hypothesis should be investigated in future studies.

Differential fuel utilisation responsible for the distinct responses of the NPY/AgRP and POMC neurons to metabolic signals has been characterised, whereas POMC neurons utilise glucose as the main fuel, NPY/AgRP neurons do not use glucose, but free fatty acids instead. This differential fuel utilisation implies two distinct and competitive mechanisms, glycolysis and ß-oxidation, in these neuronal populations. When glycolysis is elevated, ß-oxidation is inhibited and vice versa. Thus, glucose enhances POMC and reduces NPY/AgRP neuronal activity. The by-products of substrate oxidation are reactive oxygen species (ROS) that have a crucial role in the acute and the long-term regulation of feeding, satiety, and associated metabolic changes (i.e. glucose and fatty acid homeostasis). Mitochondrial ROS (mROS) is a necessary signal to initiate the response to glucose sensing. A finely controlled mROS production might be considered as an essential physiological messenger in metabolic-sensitive cells [114, 115]. Alteration of the hypothalamic glucose-sensing mechanism induced dramatic effects on energy balance correlated to abnormal redox signalling originated from mitochondrial dysfunction [116]. During negative energy balance, NPY/AgRP neurons utilise free fatty acids as fuel, but ROS levels are not increased in these cells despite increased firing and substrate utilisation. In contrast, during positive energy balance, when glucose-utilising POMC neurons are firing at high levels, ROS accumulate in these POMC cells because they do not need to be buffered [50]. Sustained ROS levels in POMC neurons seem to favour satiety. The fact that satiety is associated with the highest levels of ROS production in the POMC neurons indicates that these cells are more exposed to ROS-induced damage than NPY/AgRP neurons, which do not produce elevated ROS levels even if highly active. Thus, it seems plausible that POMC neurons are more exposed to elevated firing (positive energy balance) over time, thus leading to POMC system impairment. In contrast, since NPY/AgRP neurons are inherently able to buffer ROS, their increased activity during negative energy balance is not associated with ROS-induced degeneration [50].

**Fig. 1:**
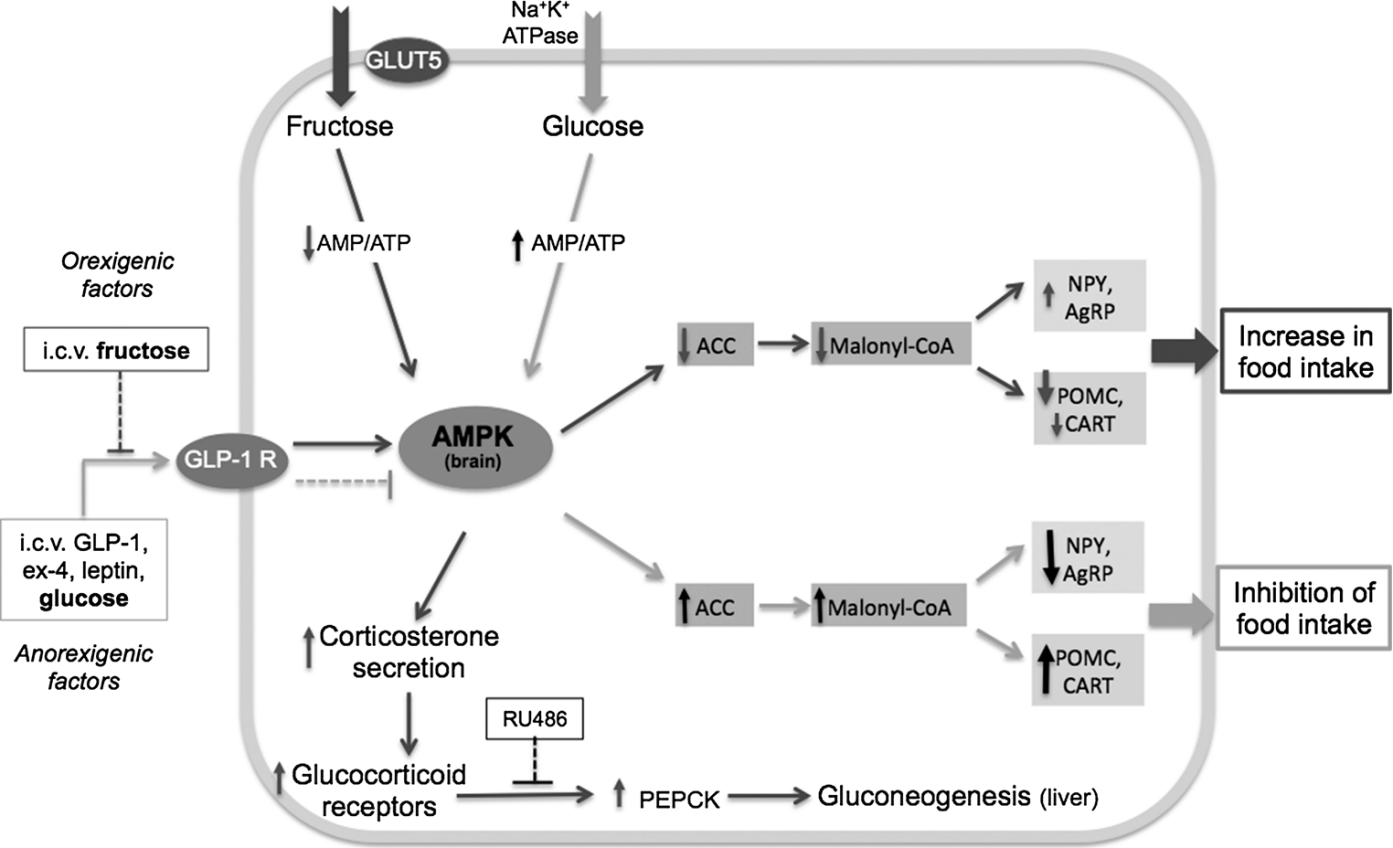
Effects of an i.c.v. injection of fructose or glucose on central neuropeptides and appetite. Glucose transport is facilitated by the Na+ gradient. Fructose transport across the membrane by GLUT5 does not need ATP. Fructose bypasses the rate-limiting step in glycolysis, which generates a decrease in AMP/ATP ratio, the phosphorylation and activation of AMPK (the cell sensor of AMP/ATP ratio) in the liver [180], and in hypothalamic neurons [50, 54]. This stimulates corticosterone secretion, activating glucocorticoid receptors followed by activation of phosphoenol pyruvate carboxykinase (PEPCK) and gluconeogenesis [181]. The activation of PEPCK induced by fructose was prevented by RU486, a glucocorticoid receptor antagonist [118]. Intracerebroventricular (i.c.v) injection of the GLP-1 receptor (GLP-1R) agonist exendin-4 (Ex-4), suppressed AMPK activity in hypothalamic cells and food intake; i.c.v fructose attenuated the anorectic effect of Ex-4, suggesting a mechanism for the increased food intake by fructose via impairment of central GLP-1R action [117]. Glucose injected i.c.v. increased ATP/AMP ratio, activated AMPK, acetyl-CoA carboxylase (ACC) and malonyl CoA, leading to decreased mRNA levels of orexigenic neuropeptides NPY and agouti-related protein (AgRP), while activating the expression of the anorexigenic peptides cocaine–amphetamine-related transcript (CART) and proopiomelanocortin (POMC). These signals suppress food intake and increase energy expenditure. Fructose i.c.v injected exerts an orexigenic effect by lowering malonyl CoA mRNA levels [54]

The role of AMPK in the regulation of food intake has been well demonstrated. Regulatory mechanisms of food intake controlled by central AMPK activity in response to an i.c.v. injection of glucose and fructose are presented in Fig. 1. These data suggested an anorexigenic effect of i.c.v. glucose injection through inactivation of AMPK, an increase in malonyl CoA, and in anorexigenic neuropeptides mRNA levels in the hypothalamus, whereas i.c.v. fructose injection resulted in the inverse effects [50, 54, 117, 118] (see Fig. 1). However, whether fructose orally ingested can also produce these effects than when administered i.p. or i.c.v. remains to be investigated.

Another potential mechanism for the fructose effects on food intake might be through the nuclear receptors liver X receptor (LXR) α and β. These receptors have been previously implicated in the regulation of carbohydrate and lipid metabolism [119] and were shown to be expressed in the hypothalamus and implicated in the regulation of food intake [120]. Free access to a diet-containing 10 % fructose for 6 weeks in glucose-intolerant rats induced a decrease in LXRβ and an increase in LXRα in the hypothalamus, but not in the hippocampus, cerebellum, or neocortex. It is possible that the specific hypothalamic increase in LXRα by fructose may trigger neurochemical and neurophysiological responses for the control of food intake and energy expenditure [120]. But further studies are needed to investigate this hypothesis.

Circulating fructose levels could possibly promote central effects in humans even if hepatic clearance of fructose is extremely efficient, given the following observations: (i) the presence of GLUT5 in the BBB and ketohexokinase mRNA, the necessary cellular machinery for fructose metabolism [57]; (ii) fructose administered i.p. can cross the BBB and trigger neuronal activation in rodents [57]; (iii) fructose administrated i.p. can be metabolised to lactate in the hypothalamus [121]. These data suggested the capacity of fructose to cross the BBB into the hypothalamus, where it could be metabolised and used as an energy source. Thus, consumption of high-fructose diets might probably have a direct effect on the brain, but no study has clearly proven this concept yet. While it is well established that glucose orally ingested undergoes facilitated transport across the BBB [122], the demonstration that fructose orally ingested can cross the BBB is still missing. Another question is how changes in feeding behaviour associated with glucose- and fructose-induced activation of brain regions observed in animals could be extrapolated to humans, especially when most of the studies investigating these effects have been performed on rodents.

### Dietary sugar sensing on brain activation and eating behaviour

New technologies are available to facilitate the translation of animal to human studies and help understanding of brain functions. One such technique is the single-photon emission computed tomography (SPECT) that provides a way to compare brain circuits implicated in the processing of oral and/or visceral (e.g. duodenal or portal) sugar signals. Boubaker et al. [123] evaluated brain activity in a juvenile pig model using SPECT following visceral nutritional stimulation. The authors found that both duodenal and portal glucose infusions activated the dorsolateral prefrontal cortex and primary somatosensory cortex. However, only duodenal glucose infusion induced the activation of the prepyriform area, orbitofrontal cortex, caudate and putamen, and the deactivation of the anterior prefrontal cortex and anterior entorhinal cortex, whereas only portal glucose infusion induced the activation of the insular cortex. These results indicated that duodenal and portal glucose infusions modulate differentially the activity of brain areas implicated in the regulation of eating behaviour, which probably explains the decrease in food intake after both stimulations [123]. Another SPECT study in pigs (Clouard et al., 2013, unpublished data) demonstrated that combined oral and duodenal sucrose sensing induced activation of brain regions involved in memory, reward processes, and hedonic identification of sensory stimuli (i.e. amygdala, dorsal striatum: caudate and putamen, and the anterior prefrontal cortex), whereas oral or duodenal sucrose sensing individually administered did not. These findings suggested that (1) the concordance between oral and visceral signals (sweet taste and calories) during sugar sensing is necessary for the onset of responses in these brain structures, or (2) the synergy between oral and visceral signals during sugar sensing is required to obtain a signal that is strong enough to trigger brain responses in these structures (Clouard, et al., 2013, unpublished data).

Positron emission tomography (PET) studies with 18-fluorodeoxyglucose (18-FDG) to measure brain glucose metabolism in normal-weight individuals reported that exposure to food cues increased metabolic activity in the orbitofrontal cortex, similar to that observed in cocaine-addicted subjects, which was an effect associated with the perception of hunger and the desire for food [124]. Functional magnetic resonance imaging (fMRI) is another technique that provides a non-invasive way to assess the effects of glucose and fructose intake, as well as of obesity, on regional cerebral blood flow (abbreviated rCBF and estimated via the blood–oxygen-level-dependent or BOLD signal) [97]. Previous studies have explored the temporal response to glucose intake or infusion using fMRI and found suppression of hypothalamic BOLD signalling after the administration of glucose to rats and humans. Obese subjects presented diminished attenuation of the BOLD signal in response to glucose ingestion compared with lean subjects, and patients with type 2 diabetes did not show any hypothalamic signal changes compared with non-diabetic patients [125]. This might suggest a reduced neuronal activation in obese relative to lean subjects, which might translate in no suppression of appetite and less rewarding signals from glucose intake leading to overeating. Cortical responses to sugars in healthy subjects as assessed by fMRI appear to be opposite between glucose and fructose infusion: increased and decreased cortical activation, respectively [126]. The suppressive effect of fructose on cortical BOLD signal occurred despite the fact that cortical-specific receptors (GLUT5) are present in low concentrations throughout the brain, where the glucose transporter GLUT3 predominates. Whether this cortical response to fructose is due to effects mediated by GLUT2 and GLUT5 carriers in the BBB or in local glial cells, due to increased osmolality, or is the indirect result of changes in the levels of peripheral neural input or metabolic intermediaries is yet to be understood. However, the overall observations suggest a major implication of fructose on changes in brain activity similar to those observed with addictive drugs, which may lead to an altered reward response to palatable food [126].

In contrast to the effects of sugars in cortical activation, their effects in other brain regions involved in food intake control, i.e. hypothalamus, appear to be opposite. Another fMRI study in humans showed that glucose, but not fructose intake, induced a marked reduction in hypothalamic BOLD signal, as well as a reduction in CBF within the thalamus, insula, anterior cingulate, striatum, and hypothalamus, i.e. brain regions that act together to sense the metabolic state of an individual and drive motivation and reward. Moreover, fructose produced a transient increase in hypothalamic activity and reduced CBF in the hippocampus, a region implicated not only in memory but also influencing emotional responses to food intake. These findings suggest that ingestion of glucose, but not fructose, initiates a coordinated response between the homeostatic and striatal networks that regulate eating behaviour [57]. In line with these data, ingestion of glucose but not fructose produced increased ratings of satiety and fullness [57]. What are the underlying mechanisms of these effects of fructose on CBF changes is a remaining question since no study has clearly demonstrated the capacity of fructose orally ingested to cross the BBB.

**Fig. 2:**
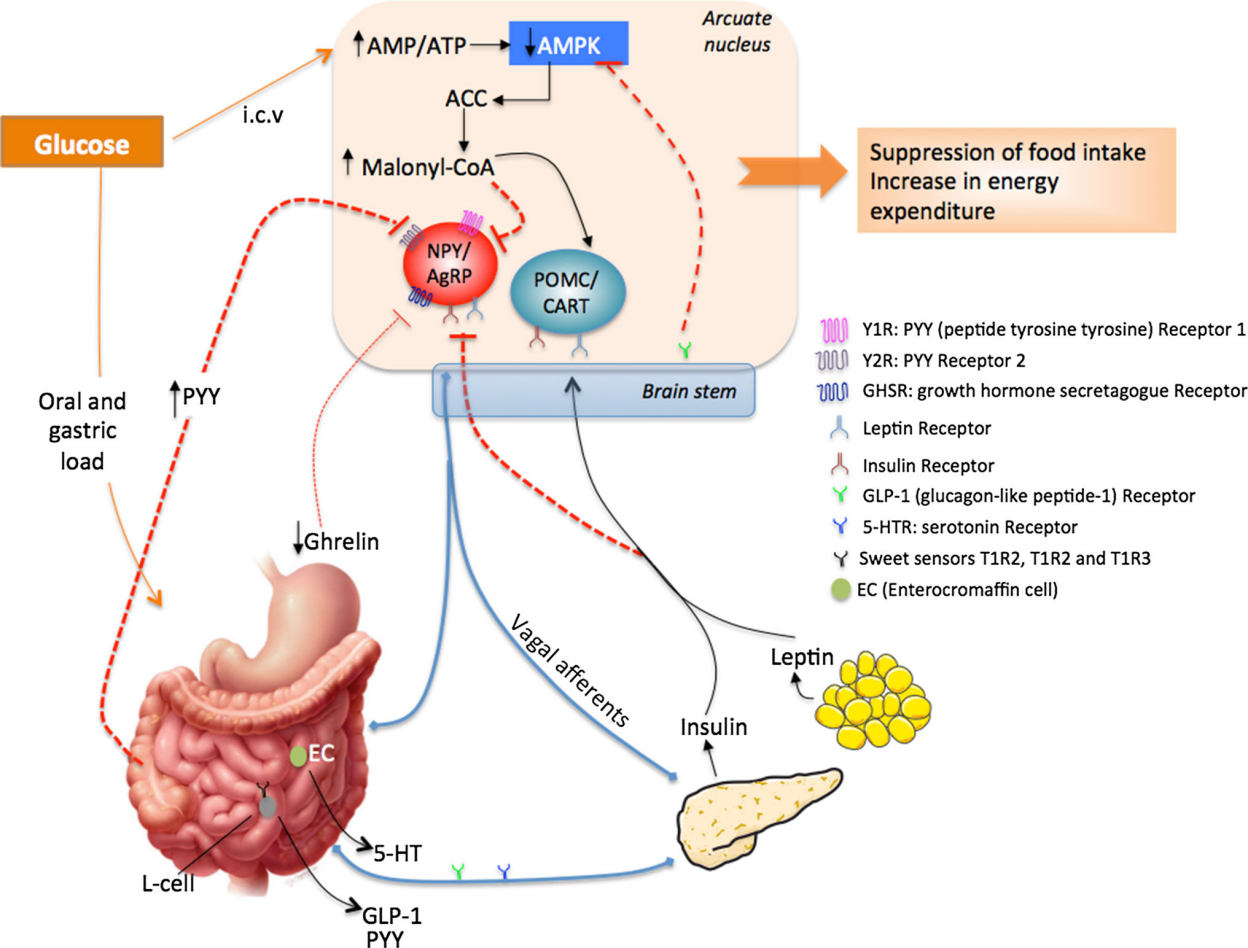
Hypothetical model of the peripheral and central effects of glucose on food intake. Luminal glucose activates vagal afferents via the release of 5-HT or GLP-1. Vagal afferents express GLP-1 and 5-HT receptors, and are implicated in the regulation of insulin secretion. Many neuronal signals are communicated via the vagus nerve to the brain stem, which relays the glucose signal to hypothalamic nuclei and then to the pertinent target cells: NPY/AgRP and POMC/CART neurons. *NPY* neuropeptide Y, *AgRP* agouti-related protein, *POMC* proopiomelanocortin, *CART* cocaine–amphetamine-related transcript, *AMP* adenosine monophosphate, *AMPK* AMP kinase, *ACC* acetyl-CoA carboxylase. *Black arrows* activation; *discontinued* *red lines* inhibition; *thin discontinued red line* weak activation [4, 51, 52, 54, 57, 58, 72, 182]

**Fig. 3:**
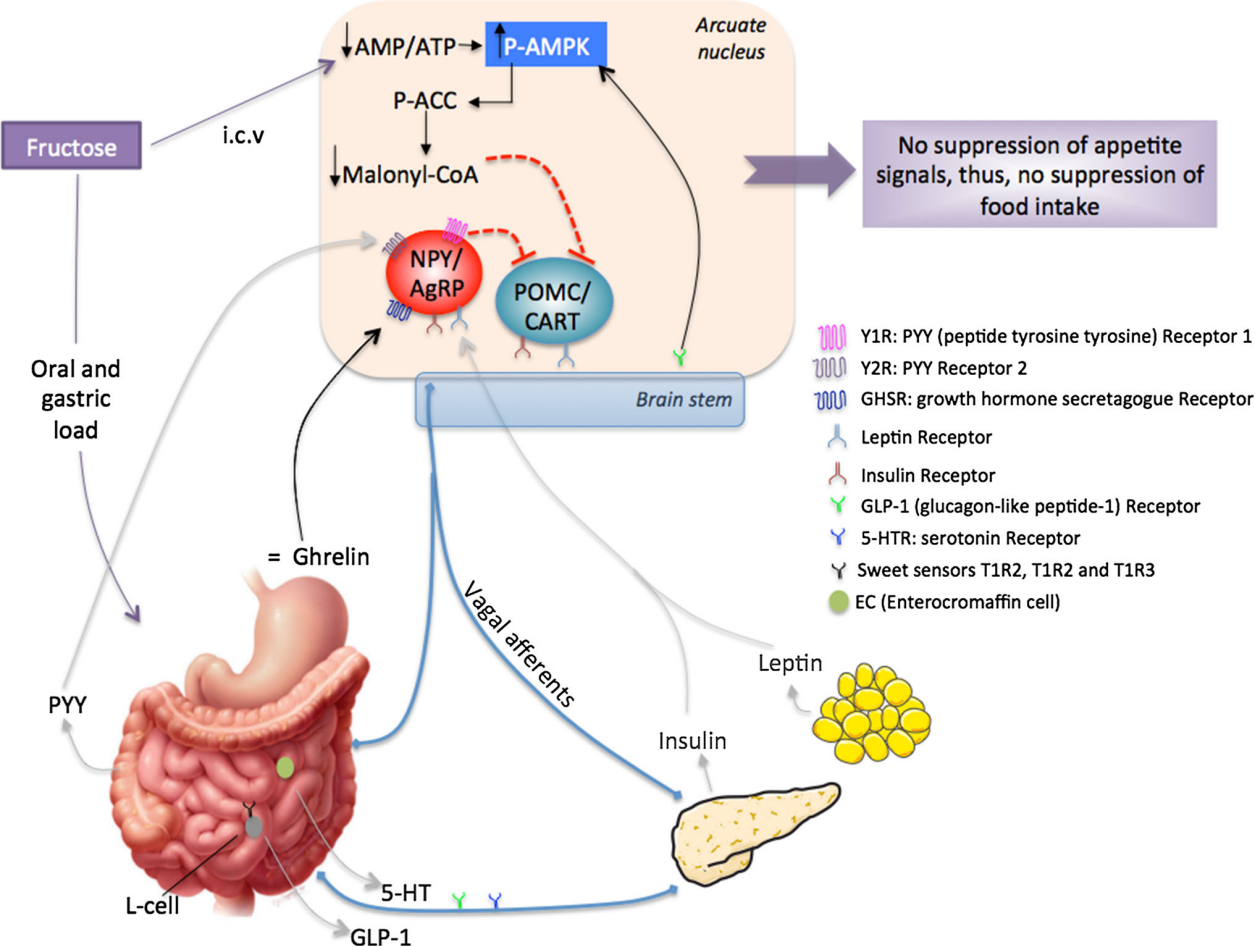
Hypothetical model of the peripheral and central effects of fructose on food intake. Luminal fructose induces weak release of 5-HT and GLP-1 from enteroendocrine and L cells, respectively, weak PYY, insulin and leptin secretion, as well as weak ghrelin suppression. Many neuronal signals are communicated via the vagus nerve to the brain stem, which relays the glucose signal to hypothalamic nuclei and then to the pertinent target cells: NPY/AgRP and POMC/CART neurons. *NPY* neuropeptide Y, *AgRP* agouti-related protein, *POMC* proopiomelanocortin, *CART* cocaine–amphetamine-related transcript, *AMP* adenosine monophosphate, *AMPK* AMP kinase, *ACC* acetyl-CoA carboxylase. *Black arrows* activation; *discontinued red lines* inhibition; *grey arrows* low secretion or low activation [4, 37, 51, 52, 54, 58, 59, 72, 182]

Figures 2 and 3 represent hypothetical and controversial models summarising the findings that support the ‘fructose hypothesis’ which postulates an orexigenic and less rewarding effect of fructose intake, compared to glucose. It presents the underlying molecular mechanisms throughout the gut–brain communication supporting this hypothesis.

## Dietary sugars and the mesolimbic dopamine system

The mesolimbic dopamine (DA) system plays a critical role in the reinforcing effects of reward and is implicated in conditions such as drug addiction and eating disorders. Dopamine is the primary neurotransmitter involved in the brain reward pathways [127] and in the reward value of sweet taste, mainly because sweet taste activates mesolimbic DA circuits involved in the mediation of natural as well as drug rewards. Mice lacking the cellular machinery required for sweet taste transduction (trpm5^−/−^) learned to prefer the postingestive effects of sucrose. These mice did not develop a preference for sucrose per se probably due to the short training sessions [128]. However, Zuckerman et al. [129] reported that trpm5 KO mice learned to prefer glucose but not fructose solutions in 24-h two-bottle choice tests. Furthermore, trpm5 KO mice developed a robust preference for sucrose solutions based solely on caloric content. Sucrose intake induced DA release and increased neuronal responses in the ventral striatum of these mice in the absence of gustatory input. These findings suggested that calorie-rich nutrients could directly influence brain reward circuits that control food intake independently of palatability or functional taste transduction [128]. Dopamine release is stimulated in the NAc by the sweet taste in the mouth. [130] It was reported that the neurochemical effects observed with intermittent sugar access are not only due to sucrose postingestive properties but also to the sweet taste of sucrose [130].

The mesolimbic DA projection from the ventral tegmental area (VTA) to the NAc is frequently implicated in reinforcement functions [127]. Data from animal models of binge-eating but with normal weight show that behavioural and neuronal consequences of bingeing on a palatable food are different from those that result from simply consuming the palatable food in a non-binge manner, i.e. physiological, neural, and behavioural effects independent of a DIO [131]. Excessive intake of palatable foods under specific conditions can produce behaviours and changes in the brain that resemble an addiction-like state, such as greater activation of the anterior cingulate cortex, the medial orbitofrontal cortex, and the amygdala, regions associated with motivation. These changes may be more closely correlated with binge-eating behaviour than they are to body weight [131].

It was recently suggested that people at risk of obesity initially show a hyperfunctioning in the gustatory and somatosensory cortices that increases pleasure perception from food, leading to increased reward, overeating, and weight gain. This overeating may induce receptor down-regulation in the striatum, increasing the likelihood of further overeating and continued weight gain. Obese versus lean humans show less activation in the dorsolateral prefrontal cortex, and greater activation of regions involved in the reward value of stimuli (striatum, amygdala, orbitofrontal cortex, and mid-insula), in attention regions (ventral lateral prefrontal cortex), and in somatosensory regions, in response to high-fat/high-sugar food images relative to control images [132]. Similarly, DIO minipigs showed deactivations in the dorsolateral and anterior prefrontal cortices and activations in the ventral posterior nucleus of the thalamus and middle temporal gyrus, compared to lean minipigs. Moreover, the anterior and dorsolateral prefrontal cortices as well as the insular cortex activity were negatively associated with body weight. These data suggested that the reduced activation of the prefrontal cortex observed in obese subjects is an acquired feature of obesity [22].

McCutcheon et al. [133] showed that DA release in the NAc core to food-predictive cues is strongly modulated by food’s characteristics. Cues paired with sucrose (the preferred reward) evoked greater DA release, than cues predicting saccharin (the non-preferred reward). This subjective preference and greater DA release could result from a difference in the orosensory qualities of the pellets or an aversion to the bitter taste of saccharin, and suggests that sucrose may more powerfully motivate behaviour [133].

The NAc, the amygdala, and the medial prefrontal cortex are implicated as central sites of action for the suppressive effects of the DA-1 receptor (D1R) and DA-2 receptor (D2R) antagonists (SCH23390 and raclopride, respectively), on sugar intake and on the expression of flavour preferences conditioned by the sweet taste of sugars. Subcutaneous administration of D1R agonist prior to food preference test reduced the intake of the regular diet and induced a strong preference for high-fat/high-sucrose food, whereas D2/D3 receptor agonist had the opposite effects. These results suggested that the DA receptor subtype is a major determining factor of the direction in which sweet food preference is modulated, in addition to the level of DA release in the NAc [134]. Subcutaneous injections of D1R and D2R antagonists substantially and dose dependently reduced the intake and preference of sucrose solutions but not saccharin solutions, suggesting that the antagonists decreased the reinforcing value of sucrose, but not saccharin, solution [135]. Systemic administration of dopamine D1R (SCH23390) and D2R (raclopride) antagonists in the medial prefrontal cortex and amygdala, but not in the NAc shell, blocked the acquisition and expression of fructose-conditioned flavour preferences in rats [136].

In contrast to the effect of intragastric glucose following bilateral injection of the D1R antagonist on blocking the acquisition of conditioned flavour preferences [137], bilateral hypothalamic injections of D1R and D2R antagonists failed to alter the acquisition of fructose-conditioned flavour preference in rats [138]. These findings suggest an important difference between flavour–flavour and flavour–nutrient-conditioned flavour preferences. Fructose intake and fructose-conditioned flavour preference appear to be more dependent on D2R activity than sucrose intake. Injections of D1R antagonist into the amygdala or NAc during training did not block fructose conditioning, but did block the acquisition of a flavour preference produced by glucose infusions [139]. Rats under a high-fat diet responded to all raclopride doses with reductions in sucrose intake, but not in fructose intake, while rats fed a high-fat and sugar diet only responded to the highest dose of raclopride, with significant reductions in fructose intake [140]. These data indicate that there is a differential involvement of D1R and D2R in flavour–flavour and flavour–nutrient preference, respectively. An explanation may include differential neural and hormonal postingestive effects exerted by fructose and sucrose. In this context, it may be possible that the high-fat diet and the high-fat and high-sugar diet altered sucrose and fructose preferences differently as a result of their differential effects on oral and gastrointestinal signals upstream the reward system [140].

### Interactions between peripheral signals and the mesolimbic dopamine system

Anatomical and functional evidence demonstrates multiple interactions between the mesolimbic DA system and peripheral signals regulating food intake. One such system is the CCK system. In an obese rat model lacking the CCK-1 receptors (Oletf), treatment with D2R antagonist raclopride, but not D1R antagonist SCH23390, showed increased potency to reduce sucrose real intake, indicating that D2R are involved in heightened increased consumption of sucrose observed in these obese rats. These findings confirm the notion that DA increases sucrose intake due to the assignment of an actual rewarding value of sucrose primarily based on its sensory stimulatory effects. They also confirm that altered DA signalling present in obesity is involved in the increased potency of sucrose palatability to maintain ingestion in obese subjects [141].

Ghrelin has recently emerged as a potent modulator of the mesolimbic dopaminergic reward pathway, suggesting a role for ghrelin in food reward. Ghrelin targets a key mesolimbic circuit involved in food and drug-induced reinforcement, i.e. DA projection from the VTA to the NAc [142]. Skibicka et al. [143] identified the VTA, a key structure in the mesolimbic reward system, as a primary target for ghrelin’s effects to increase motivation for a sweet food reward. Peripherally and centrally administered ghrelin significantly increased operant responding and incentive motivation for sucrose. Conversely, blockade of GHS-R1A (Ghrelin type 1A receptor) signalling significantly decreased operant responding for sucrose. These findings indicate that ghrelin plays an important role in motivation and reinforcement for sucrose. They suggest that ghrelin antagonists have therapeutic potential for the treatment for obesity and for suppressing sweet food overconsumption [143, 144].

Lustig [35] reviewed that chronic hyperinsulinemia may prevent DA clearance from the NAc and leptin signalling, leading to leptin resistance and increased food intake. Thus, by promoting insulin resistance and hyperinsulinemia, fructose excessive intake may alter DA neurotransmission and the hedonic response to food leading to overeating [35]. Increasing the palatability of food by the addition of sucrose undermines normal satiety signals and motivates energy intake independent of energy needs [145]. Animal models of intermittent sugar administration can induce behavioural alterations consistent with dependence, i.e. bingeing, withdrawal and anxiety, craving, and cross-sensitisation to other drugs of abuse [146].

## Dietary sugars and the endocannabinoid system

The endocannabinoid system is a lipid-signalling system composed of three non-ubiquitous receptors (CB1, CB2, and likely CB3 receptor), two endogenous ligands (i.e. anandamide and 2-arachidonoyl-glycerol, 2-AG), and the enzymatic machinery for their synthesis and degradation [147]. This system is implicated in the regulation of appetite, eating behaviour, and body weight homeostasis at both peripheral and central levels. In the brain, the endocannabinoid system appears to control food intake mainly at three functional levels, i.e. the hypothalamus, the dorsal vagal complex, and the limbic system, by affecting satiety signals and interacting with brain reward pathways [148, 149]. Previous studies have revealed the ability of marijuana, or of its main psychoactive component Δ^9^-tetrahydrocannabinoid (Δ^9^-THC), to induce not only hyperphagia, but also to increase the desire to consume highly palatable food and to impact food selection concomitantly [150].

Endocannabinoids have been implicated in the regulation of consumption of palatable food, sugar in particular. CB1 receptor antagonist (SR 141716, also known as Rimonabant, an anorectic anti-obesity drug) resulted in reduced body weight and appetite for sweet foods and drinks [151]. Following low oral doses of Δ^9^-THC (0.25 and 0.40 mg/kg), there was a dose-dependent increase in preference for palatable food and sucrose intake in rats. Similarly, administration of Δ^9^-THC (0.5, 1.0, and 3.0 mg/kg), anandamide (1.0 and 3.0 mg/kg), and 2-AG (0.2, 1.0, and 2.0 mg/kg) substantially increased the number of licks of 10 % sucrose solution, due to increased bout duration rather than bout number, whereas administration of CB1 antagonist SR141716 significantly decreased total licks [152]. Rimonabant also specifically reduced sucrose, alcohol, and sweet food intake in rats and marmosets [148]. Endocannabinoids (anandamide and 2-AG) peripherally administered selectively enhanced gustatory nerve (chorda tympani) responses and electrophysiological responses of taste cells, located on the anterior tongue innervated by the chorda tympani nerve, to sweeteners (i.e. saccharin, glucose, and sucrose) in mice. These sweet-enhancing effects of endocannabinoids were mediated by CB1 receptors, which were coexpressed in taste cells with the sweet receptor subunit T1R3 in taste cells. Indeed, endocannabinoid administration also increased T1R3 taste cells responses to sweeteners [153]. These effects remain to be explored with fructose stimulation. However, since no differences were previously found in gustatory nerve (chorda tympani) responses to glucose, sucrose, and fructose in pigs [154], one might speculate that endocannabinoids may exert similar degree of taste enhancement sensitivities between these three sugars. But this hypothesis remains unclear since neural responses do not always predict functional sensitivity; moreover, the observed responses do differ at various stimulus concentrations, and it is unclear how endocannabinoids would interact with this. Intraperitoneal administration of endocannabinoids to wild-type mice selectively enhanced gustatory nerve responses and electrophysiological responses of taste cells, located on the anterior tongue innervated by the chorda tympani nerve, to sweet compounds (sucrose, saccharin, and glucose). These sweet-enhancing effects of endocannabinoids were mediated by CB1 receptors, which are coexpressed in taste cells with the sweet receptor component T1R3 [153].

Consumption of fructose solution in combination with standard rat chow resulted in increased mRNA levels of CB1 in the rat hypothalamus [7]. Intake of sucrose, glucose, and fructose solutions during 7 days affected the mRNA expression of the majority of enzymes involved in the synthesis and degradation of anandamide and 2-AG, in rats [10]. Fructose solution increased mRNA levels of fatty acid amide hydrolase (FAAH) (involved in anandamide degradation), compared to water, glucose, and sucrose solutions. This suggests that fructose intake might induce an overproduction of anandamide and that an up-regulation of this enzyme is necessary to maintain normal levels of anandamide. The three sugar solutions induced a down-regulation of phospholipase C (involved in anandamide synthesis). This may suggest an attempt to maintain anandamide at physiological levels during periods of high-sugar consumption irrespective of the nature of the sugar. Monoglyceride lipase (MGLL), the main enzyme involved in 2-AG degradation, was also down-regulated by the three sugar solutions compared to water intake. This would suggest that 2-AG is degraded less readily in rats drinking sugar solutions than in water drinking rats. However, only fructose intake increased mRNA levels of diacylglycerol lipase 1β (involved in 2-AG synthesis), suggesting that more 2-AG is being produced [10]. However, the interpretation of these results is conflicting, given the simultaneous increase or decrease in enzymes involved in the synthesis and degradation of endocannabinnoids, respectively, induced by the sugar solutions. Besides, neither protein levels nor actual concentrations of endocannabinoids were measured, making it difficult to draw a conclusion. Further studies are needed to understand the implications of these results. Cani et al. [155] demonstrated that a diet-induced obesity (DIO) by excess dietary lipid intake is associated with altered expression of CB1 mRNA, higher plasma endocannabinoids, or increased adipose tissue endocannabinoid synthesis. Blockade of CB1 receptor improves the gut barrier and reduces metabolic endotoxemia, by a mechanism independent of eating behaviour, suggesting a control of gut permeability by CB1 receptors through interactions with gut microbiota [155]. It is possible that fructose could also modulate the intestinal endocannabinoid system by a similar mechanism, but future studies are needed to investigate this hypothesis.

Finally, endocannabinoids have been proven to interact with brain reward pathways in a manner similar to other reward-enhancing drugs. Therefore, the endocannabinoid system might affect eating behaviour through the modulation of the reward circuit [148]. Which are the effects of the different dietary sugars on the endocannabinoid system and their interaction with brain reward pathways to affect eating behaviour is a question that remains incompletely understood and needs further investigation. As presented in the previous section on the DA system, different types of sugars differentially modulate this system. Therefore, it is possible that these differences might also be observed in the endocannabinoid system and this requires further investigation.

## Dietary sugars and the opioid system

Opiodergic neurotransmission within the brain reward circuit mediates hedonic aspects of sweet-palatable foods [8]. Chronic suppression of the endogenous µ-opioid receptor signalling in the nucleus accumbens (NAc) shell and core significantly attenuates the development of a DIO by reducing the intake of palatable, high-sugar foods in rats [156]. Opioid antagonism in the NAc is associated with a reduction in sweet food preference and sucrose intake, and weakens hedonic properties of sucrose and motivation for sucrose [156–158]. The latter (‘wanting’) may be attributable to the decrease in ‘liking’ (hedonic properties) [158]. Conversely, stimulation of µ-opioid signalling in the NAc increases sucrose intake and motivation [159].

The consumption of sweet tastants results in neurochemical changes within the brain, which may reflect a shift in opioid-mediated responses. Sucrose and glucose intake paired with opioid receptor antagonism (naloxone) induced an increase in the number of c-Fos-positive nuclear profiles [160] and an elevation in opioid µ-1 receptor binding in the cingulate cortex, hippocampus, locus coeruleus, and accumbens shell, associated with the presence of opiate withdrawal-like symptoms, such as teeth chattering [161]. These results suggest that ingestion of sucrose and glucose induces neurochemical changes within the opioid brain circuitry. Opioids support a drive to consume sugar, and this mechanism is mainly dependent on their ability to act through the reward system. Similar to the reports in the cocaine studies for drug euphoria and craving, sweet liking may increase with the dose while sweet wanting may not [162].

Overall, opioid signalling, particularly through its µ-receptor in the NAc, is involved in the expression of reward behaviours induced by the consumption of sweet-palatable foods and may be involved in the development of DIO. However, the vast majority of these studies, in both animals and humans, regarding opioids and sugar intake have used sucrose as the source of sugar. One of the few studies using both glucose and fructose was performed by Bernal et al. [163]. They found that rats develop strong preferences for flavours paired with the sweet taste of fructose or the post-oral nutrient effects of glucose. Opioid antagonism at the NAc shell and core did not block sugar-conditioned flavour preference at any dose and with neither glucose nor fructose solutions [163]. However, the authors did not directly compared to glucose versus fructose stimulus so there is no clear evidence about possible differences between these sugars following opioid antagonism in the NAc. Given the high amounts of fructose currently consumed in Western diets, it would be interesting to directly compare the effects of free fructose and glucose to those observed with sucrose on the opioid system. Given the observations of the effects of fructose intake on appetite, its higher palatability compared to glucose, and its differential effects at both peripheral and central levels, including the DA system, one might speculate that fructose could induce more profound effects on this system than sucrose or glucose. However, since fructose may not cross the BBB at typical intake levels, this hypothesis seems unrealistic. Further research directly comparing the effects of sucrose, glucose, and fructose is needed to investigate this hypothesis.

## Controversial findings

Several pieces of evidence have led to the assumption that fructose excessive intake may be responsible for the increasing prevalence of obesity since the last decades. This has stimulated research aiming at understanding the underlying mechanisms of this fructose-induced obesity. For example, epidemiologic and experimental evidence indicates that a greater consumption of sugar-sweetened beverages with HFCS is, in fact, associated with weight gain and obesity, and that HFCS accounts for 40 % of caloric sweeteners used in the United States [13, 16, 17, 164, 165]. In addition to these epidemiological data, evidence has shown that fructose induces smaller increases in insulin, leptin, and other satiety peptides compared to glucose [37, 55–59]. This suggested an endocrine mechanism by which fructose might induce greater food intake and weight gain than glucose. However, while it is true that fructose intake in the form of HFCS makes up a significant proportion of energy intake in the Western diet [13, 164], it is also true that this increase in fructose intake is necessarily associated with an increase in total energy intake and in glucose (from HFCS). Besides, equal amounts of glucose and fructose are necessary for maximal fructose absorption in humans [166]. This makes questionable the effect of fructose per se for increasing food intake, inducing weight gain and metabolic diseases [16]. In fact, there are some well-controlled studies showing divergent findings in this regard that are important to discuss here given the extended great concern regarding the fructose-induced obesity.

Lindqvist et al. [7] found no differences in terms of food intake, PYY, and leptin serum levels following 2-week intake of sucrose, glucose, or fructose solutions in rats. Moreover, these authors found that the fructose-drinking group had the smallest increase in food intake, probably attributed to the lowest intake of fructose solution. This was attributed to the fact that fructose is sweeter than sucrose and glucose [7], and it may also be attributable to the short period (2 weeks) of sugar solution intake. In addition, no difference was found in energy intake and weight gain following 50-day intake of sucrose, glucose, or fructose solutions, but body adiposity increase was greater with sucrose than with fructose solutions [70]. In humans, no differences in terms of energy intake, satiety, and energy compensation, nor in plasma glucose, GLP-1, insulin, and ghrelin release were found following acute ingestion of preload drinks containing sucrose or HFCS (1.5 MJ) [167]. This lack of difference in satiety was found despite different biochemical properties (leading to different transport across the gut epithelium and thus different transit time) as well as different mechanisms underlying satiety between sucrose- and HFCS-containing drinks [64, 110, 168]. Therefore, more studies are needed to clarify these discrepancies.

Glendinning et al. [169] investigated in four strains of mice given free access to sugar solutions and showed that sucrose promoted more overeating, resulting in increased weight gain and adiposity compared to fructose, regardless of mouse strain. Moreover, all strains licked more avidly the sucrose than the fructose solutions. These authors reported as well that mice and rats consume less fructose than isocaloric sucrose [169]. Fructose orally ingested or intraduodenally infused induced insulin release and inhibited food intake more than glucose in rats and humans [7, 170, 171], whereas another study found no difference between these sugars orally ingested on food intake [172].

Sclafani and Ackroff [173] reported that 16 % glucose intragastric infusion condition a strong flavour preference in mice, whereas fructose and galactose infusions failed to do so. The latter findings are opposed to other findings, suggesting that fructose is a weaker elicitor of satiation signals [54, 62] and may have a more rewarding effect probably due to its higher sweetness than glucose [174]. The only rodent study that appears in the literature reporting fructose-induced weight gain more than sucrose-fed mice used a 15 % fructose solution or a 10 % sucrose solution, which invalidates the findings [67]. White [175] recently reviewed that many animal studies have used extremely high-fructose doses, or altered the usual glucose-to-fructose ratio that are not predictive of typical human diets, leading to abnormal metabolism. He exposed as well that (i) the increased energy intake per capita coupled with insufficient compensating exercise is a more consistent explanation to the obesity epidemics; (ii) there has been no positive correlation between fructose intake and increasing rates of obesity; (iii) consumption of added sugars has not increased, but actually decreased for more than a decade; and (iv) all sources of fructose in human diets contain comparable amounts of glucose, and glucose is the dominant sugar in the human diet (5 times more glucose than fructose). Besides, fructose is rapidly metabolised in the liver to glucose. The fructose hypothesis is refuted by studies using real-world fructose exposures showing no differential effects versus control, and cause-and-effect evidence of adverse effects is lacking at typical human exposure levels and patterns [175]. Sun et al. [176] analysed the intake patterns of > 25,000 subjects in the NHANES 1999–2006 databases and found that daily fructose intakes with the American diet averaged 9 % of daily intake, that fructose is rarely consumed solely or in excess over non-fructose sugars, and that fructose and non-fructose sugar ordinary intake was not positively associated with indicators of metabolic syndrome, uric acid, or BMI.

A metaanalysis by Sievenpiper et al. [177] reported the effects of fructose on body weight in controlled feeding trials. They found that fructose has no effect on body weight in isocaloric trials (637 participants) compared to isocaloric diets containing a non-fructose sugar. In contrast, high doses of fructose in hypercaloric trials (119 participants) induced weight gain. The effect of fructose-induced body weight gain in hypercaloric trials may have been due to excess energy intake rather than fructose itself because (i) weight gain is similar to that which would be predicted with consumption of a 2,000-kcal diet with similar amount excess energy; (ii) high-precision estimates of energy expenditure, fat, and carbohydrate oxidation using whole-body calorimetry showed no differences among fructose, glucose, or sucrose [178]. Taken together, these data suggest that an excess energy may be a more important factor for weight gain than the type of sugar.

Concerning some of the central effects, both oral intake of glucose and fructose solutions during 2 weeks produced a down-regulation of POMC mRNA levels [7]. Processing of POMC by pro-hormone convertases results in the production of α-MSH, which suppresses feeding, and ß-endorphin, which stimulates it. Thus, POMC mRNA decrease, together with a decrease in ß-endorphin, may indicate the down-regulation of a potent suppressor of food intake and less rewarding signals through the opioid pathway by sugar solutions. mRNA levels of NPY were also reduced by the consumption of both sugar solutions. NPY is a potent stimulator of feeding, especially sugar intake. Thus, the observed reduction in NPY mRNA levels in this study may attempt to balance for calorie overconsumption. Despite the down-regulation of hypothalamic NPY and POMC mRNA, there was no reduction in hyperphagia induced by the consumption of sugar solutions [7].

Taken together, the ‘fructose hypothesis’ remains controversial, and a cause-and-effect association between fructose intake and the metabolic syndrome and obesity has not been clearly confirmed yet. Thus, there is a need for more research on fructose with experimental designs based on physiological conditions so that a consensus could be established.

## Conclusions and perspectives

The aim of this review was to critically discuss the effects of dietary sugars at both central and peripheral levels. Based on the current findings, diverse hypotheses were postulated all along the review sections with two main goals: (i) to open new perspectives for future research that may contribute to our understanding of current data with a special focus on fructose, and (ii) to clarify controversial findings in order to advance in the establishment of a consensus concerning the differential effects of the main dietary sugars found in humans diets at both peripheral and central levels.

In summary, the reports presented here suggest differential effects of glucose and fructose at multiple levels. Contrary to glucose, excessive fructose intake may provoke metabolic disturbances, such as an increased gut permeability, low-grade inflammation, NAFLD, insulin resistance, and dyslipidaemia. Through luminal gut detection and following the activation of sweet taste receptors, glucose triggers the secretion of peripheral anorexigenic peptides, i.e. insulin, leptin, GLP-1, PYY, and suppression of orexigenic peptides (e.g. ghrelin) that activate vagal pathways and act on brain target regions controlling appetite (e.g. the arcuate nucleus of the hypothalamus and the dorsal vagal complex), thus leading to appetite suppression and reward response. Glucose may also directly induce its effects on appetite suppression by crossing the BBB, where it suppresses AMPK activity, an effect that stimulates neuronal activity of POMC/CART expressing neurons, which contributes as well in the satiety response. Fructose may have different effects on the secretory profile of appetite peptides and neuropeptides, leading to reduced appetite suppression as well as an indirect effect on the reward response, i.e. through a deficient stimulation of leptin and insulin secretion, hormones implicated in the rewarding effects of palatable food, fructose may provoke a deficient reward response leading to overeating. However, most of these results were obtained from rodents and using extremely high doses of fructose far from the typical human diets. Other studies found small or no differences between glucose, sucrose, and fructose in appetite peptide secretion, food intake, and weight gain. These discrepancies may be due to differences in species, metabolic phenotype, experimental approaches, form of administration (peripheral or central infusions), in the form of solutions or added in the diet, doses, duration of exposure, and experimental diet compositions. These differences make difficult to interpret and find a definite conclusion on these effects. Several clues and hypothesis were proposed for future research aiming at clarifying these controversial findings. Besides, fructose is rarely consumed isolated in the diet, but rather in the form of HFCS or sucrose, or consumed along with glucose. In fact, luminal glucose enhances fructose absorption. Therefore, no definite conclusions could be established for giving any nutritional recommendations to suppress or reduce fructose from the human diets. Overall, data obtained from well-controlled studies in pigs or rodents, and epidemiological studies in humans, suggest that it is the association between fructose and other components in the diet, such as fat, cholesterol, and other dietary sugars, as well as total caloric intake coming from dietary sugars, responsible for the metabolic effects and weight gain, rather than fructose intake per se. Given the difficulty to perform controlled studies in humans for ethical reasons, a valuable approach may be through the use of the pig model that has been shown to present greater similarities to humans than smaller animals (e.g. cats and rodents).

At the central level, consistent evidence has suggested the capacity of fructose to induce changes in neuropeptides or brain activity, with a resulting decrease in the satiety response. However, there is no clear evidence of the capacity of fructose orally ingested to cross the BBB. Thus, it seems unlikely that fructose could directly induce changes in brain appetite peptides to produce its effects on satiety. Fructose is partially (12 %) metabolised in the gut during absorption, and the liver and kidneys rapidly metabolise the remaining fraction. This leads to very low fructose plasma concentrations and during a very short time, as well as the low GLUT5 affinity for fructose and low GLUT5 concentrations in the BBB, and the possibility of a fructose malabsorption when ingested in high doses. These factors make unlikely that fructose could cross the BBB and induce significant effects on the brain. Contrary to the well-established direct central effects of glucose on energy homeostasis and food intake through central glucose-sensing mechanisms, the fructose effects on eating behaviour are more likely to be exclusively through indirect mechanisms, i.e. via activation of T1R2/T1R3-sensing mechanism in the mouth and gut, as well as intestinal glucose transporters. This activation triggers the secretion of appetite peptides that may affect thereafter brain neuropeptides involved in appetite control, as well as the activation or deactivation of brain regions involved in appetite and reward. Therefore, fructose may indirectly influence appetite and reward through changes in the levels of peripheral neural input or metabolic intermediaries modifying the activation of brain regions implicated in appetite and reward. While fructose effects on the reward circuitry seem to be consistent when administered i.c.v. or intragastrically, more studies are needed to confirm these effects following fructose orally ingested.

This review presented consistent evidence showing the implication of the endocannabinoid, opioid, and mesolimbic dopaminergic systems in the modulation of sweet taste reward and in the development of preferences for sweet taste that may lead to aberrant eating behaviours. This addictive-like condition could be explained by a desensitisation of the reward pathways. However, most of these studies used sucrose as the source of sugar in the experiments, thus making it impossible to separate the specific effects of glucose and fructose or their interaction. The apparent parallel increases between fructose intake and obesity development make necessary more research to elucidate possible differences between glucose, sucrose, and fructose on the reward circuitry. There may be a relationship between satiety signals and reward signals. For example, the satiety hormones insulin and leptin are implicated in the reward effect from palatable food. In this regard, a hypothesis was postulated arguing that if fructose induces lower leptin and insulin secretions, it may also induce less satiety effect and a less rewarding effect compared to glucose intake, and therefore an increase in food intake to compensate for this lack of reward from food. If this were true, then fructose would be more addictive than glucose. However, the opposite may also be plausible: if fructose is less rewarding than glucose, it may not stimulate excessive intake, as is the case of several studies reported in the ‘Controversial findings’ Section. Therefore, these hypotheses should be further explored. This may be another angle for weighing detrimental effects of sugars and thus should be investigated in future studies.

We presented results showing that sucrose intake induces DA release and increases neuronal activity in the brain reward circuitry. Dopamine release is stimulated in the NAc by the sweet taste in the mouth and by the postingestive actions of sugars. Considering that fructose is sweeter than glucose and sucrose for humans, one might hypothesise that fructose could induce greater DA release compared to glucose and sucrose, which may lead to increased reward from food, increased food intake, and aberrant eating behaviours. However, this hypothesis remains controversial since calorie-rich nutrients (i.e. sucrose) can directly influence brain reward circuits that control food intake independently of palatability or functional sweet taste transduction. On the other hand, most of the studies comparing the effects of intermittent and/or excessive sugar intake with the effects of addiction to drugs on the dopamine system have used sucrose as well, and very few have used glucose; to our knowledge, none has compared fructose versus glucose on the characteristics of addiction (e.g. escalation of intake [179]). It was until recent years that researchers began to compare the effects of DA receptor antagonists on the expression of fructose-conditioned preferences compared with sucrose. These studies have mainly found that ‘fructose intake and fructose-conditioned flavour preference appear to be more dependent on D2R activity than sucrose intake’ (e.g. [136, 138, 139]). However, the physiological implications of these results still remain to be clarified. Therefore, future studies should directly compare DA release levels, DA transporter expression and DA receptor expression patterns following ingestion of glucose, sucrose, and fructose. Concomitantly, these studies should also measure appetite peptide secretion levels, neuropeptides, brain activity of regions implicated in appetite and reward, and feeding behaviour tests (e.g. food choice, eating microstructure, operant conditioning, and progressive ratio). This integrated approach may clarify the possible links between satiety and reward effects induced by the ingestion of different dietary sugars. While some recent evidence exists showing a differential effect between glucose and fructose on the function of brain regions implicated in appetite and food reward, this concept needs to be confirmed in humans and a non-rodent animal, under controlled experimental conditions and using a physiological fructose intake. An additional question that should be addressed in future studies is which are the underlying mechanisms leading to fructose effects on brain functions, considering that this sugar might not be able to cross the BBB and directly produce these observed effects. In this regard, several hypotheses were presented here that may contribute to address this question in future studies.

Future research should therefore focus on resolving the apparently inconsistent findings, suggesting that excessive fructose intake may promote adverse effects at both peripheral and central levels to a greater extent than those provoked by glucose or sucrose. There is a particular need to integrate the metabolic, behavioural, and neurological effects of these sugars. An approach combining behavioural (e.g. progressive ratio) and metabolic (e.g. plasma and protein levels of peptides and neuropeptides) measurements, PET, and fMRI imaging, together with the use of an animal model closer to humans (i.e. the pig), would contribute to an improved understanding of the complexity of the development of diseases induced by dietary sugars.
